# Capsule Endoscopy in Patients with Implantable Electromedical Devices is Safe

**DOI:** 10.1155/2013/959234

**Published:** 2013-04-03

**Authors:** Lucinda A. Harris, Stephanie L. Hansel, Elizabeth Rajan, Komandoor Srivathsan, Robert Rea, Michael D. Crowell, David E. Fleischer, Shabana F. Pasha, Suryakanth R. Gurudu, Russell I. Heigh, Arthur D. Shiff, Janice K. Post, Jonathan A. Leighton

**Affiliations:** ^1^Department of Medicine, Division of Gastroenterology, Mayo Clinic, 13400 East Shea Boulevard, Scottsdale, AZ 85259, USA; ^2^Division of Gastroenterology and Hepatology, Mayo Clinic, Rochester, MN, USA; ^3^Division of Cardiology, Mayo Clinic, Scottsdale, AZ, USA; ^4^Division of Cardiology, Mayo Clinic, Rochester, MN, USA

## Abstract

*Background and Study Aims*. The presence of an implantable electromechanical cardiac device (IED) has long been considered a relative contraindication to the performance of video capsule endoscopy (CE). The primary aim of this study was to evaluate the safety of CE in patients with IEDs. A secondary purpose was to determine whether IEDs have any impact on images captured by CE. *Patients and Methods.* A retrospective chart review of all patients who had a capsule endoscopy at Mayo Clinic in Scottsdale, AZ, USA, or Rochester, MN, USA, (January 2002 to June 2010) was performed to identify CE studies done on patients with IEDs. One hundred and eighteen capsule studies performed in 108 patients with IEDs were identified and reviewed for demographic data, method of preparation, and study data. *Results.* The most common indications for CE were obscure gastrointestinal bleeding (77%), anemia (14%), abdominal pain (5%), celiac disease (2%), diarrhea (1%), and Crohn's disease (1%). Postprocedure assessments did not reveal any detectable alteration on the function of the IED. One patient with an ICD had a 25-minute loss of capsule imaging due to recorder defect. Two patients with LVADs had interference with capsule image acquisition. *Conclusions. *CE did not interfere with IED function, including PM, ICD, and/or LVAD and thus appears safe. Additionally, PM and ICD do not appear to interfere with image acquisition but LVAD may interfere with capsule images and require that capsule leads be positioned as far away as possible from the IED to assure reliable image acquisition.

## 1. Introduction

Wireless video capsule endoscopy (CE) had been adopted over the last decade as the procedure of choice for visualizing the small intestinal mucosa, especially in the evaluation of obscure gastrointestinal bleeding (OGIB) [1–3]. Other additional indications that have been validated are evaluation of intestinal lesions related to nonsteroidal anti-inflammatory drugs and familial polyposis [4]. It is also often used after conventional endoscopic evaluation to detect intestinal lesions in patients with Crohn's disease or suspected celiac disease [4]. This remarkable device consists of a lens, battery, light source, complementary metal-oxide semiconductor (CMOS) chip, and a transmitter. The development of the CMOS chip has allowed CE to have greater light sensitivity and require less energy than previous technology. As the capsule traverses the small intestine and is propelled by peristalsis, it transmits the acquired images via a digital radiofrequency communication channel to the data recorder that a patient wears strapped to his/her waist. This portable data recorder consists of antenna (sensor array) carried in proximity to the body, a receiver, and memory that permits accumulation of data during this examination.

The presence of an implantable electromechanical cardiac device (IED) has long been considered a relative contraindication to the performance of CE [5]. This is due to a potential interaction between the capsule (digital radiofrequency communication is at 434 MHz) and the IED from overlapping bandwidth utility [6]. This effect has its basis in the observation that radiofrequency ablation has been documented to interfere with the function of pacemakers during cardiac ablation [7]. In a small study of 23 patients with pacemakers, 52% of the devices (*N* = 12) malfunctioned and five had circuitry failure [7]. This had the unfortunate consequence that seven patients had to undergo reimplantation of a new pacemaker after removal of the malfunctioning one. There is a concern that electromagnetic interference may occur even when the device is not in direct contact with the source, for example, electrocautery devices, cell phones, and electronic surveillance systems [6, 8, 9]. 

 However, it is important that this potential concern for interference be more completely addressed and understood since this problem led the US Food and Drug Administration (FDA) and the manufacturer of the capsule (Given Imaging, Yokneam, Israel) to adopt guidelines that made IEDs a relative contraindication to the performance of CE [10]. This restriction is prohibitive of the benefits of CE as many of the patients requiring capsule studies are elderly individuals who often have implantable devices and also require evaluation for occult gastrointestinal bleeding. Previously, our group had examined the safety of pacemakers (PM) and left ventricular assist devices (LVADs) in limited number of patients and found them to be safe [10, 11]. These initial studies prompted this current larger study.

The primary aim of this study was to evaluate the safety of CE in patients with IEDs. A secondary purpose was to determine whether IEDs have any impact on images captured by CE. 

## 2. Methods

 A retrospective chart review of all patients who had a capsule endoscopy at Mayo Clinic in Scottsdale, AZ, USA or Rochester, MN, USA (January 2002 to June 2010) was performed to identify CE studies done in patients with IEDs. One hundred and eighteen capsule studies performed in 108 patients with IEDs were identified. These records were reviewed for demographic data, methods of preparation, and study completion. Most patients followed the standard protocol of remaining without food after midnight and ingesting a two-liter polyethylene glycol (PEG) preparation the night before the examination. Rochester patients did not undergo preparation with PEG and early in the study period laxative preparation for CE was not required in Scottsdale as well (*N* = 41). The patients were allowed a regular diet the day before the examination. On the day of the examination, they were allowed to take medications two hours before the test.

 All patients underwent examination in the hospital setting to enable cardiac monitoring. A standard electrocardiogram (EKG) was obtained for each patient and PM and implantable cardiac defibrillators (ICD) functions were interrogated at baseline. Cardiac rhythm was monitored during CE via Holter monitor or telemetry. The Given Diagnostic Imaging System (Yokneam, Israel) was used for CE. The capsule endoscope was activated and then swallowed by the patient with a glass of water. Selected cases had the capsule placed endoscopically because of potential problems with gastric emptying or because of prior surgery. Patients were permitted to drink 2 hours after placement of the capsule and were permitted solid food 4 hours after placement. No strenuous activity was allowed but patients were encouraged to ambulate if they were not bed bound. The sensor array and data recorder were removed after 8 hours. 

 Upon completion of the exam, the endoscopy nurse or technician transferred the accumulated data in the recorder via a high-capacity digital link to a computer workstation for interpretation. The workstation is a modified, standard personal computer that is intended for off-line storage, interpretation, and analysis of the acquired data and for generating reports. After the CE procedure, IED function was checked again by cardiology staff. All capsule results were reviewed by two investigators (L. A. Harris, S. L. Hansel) to determine demographics, indication for capsule endoscopy, and the quality of the study as well as whether or not the capsule reached the cecum. In addition, charts were reviewed for cardiac device information which included the type of device, manufacturer, date of implantation, pacer programming, atrial or ventricular capture threshold, brady/tachy parameters, and monitor findings. The Mayo Clinic Institutional Review Board (IRB) reviewed and approved this study.

The Given Diagnostic Imaging System (Yokneam, Israel) was used for capsule endoscopy. This system is composed of three subsystems—an ingestible capsule, a recorder, and a workstation. The ingestible, disposable capsule (M2A) is 11 × 26 mm and consists of four light emitting diodes, a CMOS chip, a lens, 2 batteries, a transmitter, and an antenna. During natural peristaltic propulsion through the digestive tract, the capsule endoscope acquires the video images at a rate of two pictures per second and about 50,000 images are acquired over the eight-hour period of a normal study. The hemispheric lens has a 140° field of view and magnification is in a ratio of 8 : 1. The acquired images are transmitted via a digital radiofrequency communication channel to the data recorder unit located outside the body. The portable data recorder consists of the sensor array (antenna), a receiver, and a memory device (data recorder) that allows for the acquisition of data during the exam. The sensor array is a series of adhesive pads applied in a standard manner to the abdomen or worn as a belt around the waist. The sensor array was connected to the data recorder and battery pack, which is fit in a belt worn around the patient's waist.

## 3. Results

 One hundred and eighteen capsule study reports in 108 patients were reviewed. In eight patients, the capsule study was repeated because of multiple episodes of bleeding. Sixty-three percent of the patients were male and the average age was 72.9 ± 10.6 years old. The vast majority of patients had a permanent PM (63%), followed by an ICD with or without PM (25%). Twelve percent of the patients had an LVAD. Patient demographics and CE study indications are summarized in Table 1.

 Most CE studies were performed for the evaluation of OGIB (77%) or anemia (14%). Other indications were abdominal pain (5%), celiac disease (2%), diarrhea (1%), or Crohn's disease (1%). The quality of the preparation was fair to excellent in 52% and the capsule reached the cecum in 67% of the patients.

 Pacemaker information is summarized in Table 2. Generally, PMs are of 3 types, classified according to the number of chambers involved (single (atria versus ventricle) or dual chamber) and their basic operating mechanism (on demand or automatic rate related). The majority of the pacemakers were dual chamber paced, dual chamber sensed, dual response, rate-modulated—DDDR (50%) or ventricular paced, intrinsic ventricular rhythm—VVI/VVIR (31.3%). Medtronic and Guidant were the primary PM manufacturers, accounting for almost 80% of the PM used. Pre- and postinterrogations of all of the devices failed to reveal any significant arrhythmias in any of the patients. IED functions and programmed parameters were not altered and no other complications were noted. Additionally, the patients did not complain of any problems or symptoms. 

 Postprocedure analysis of the capsule studies revealed that there was no interference on the implantable cardiac device. One patient with ICD had 25-minute loss of capsule imaging corrected by replacing the capsule device recorder. Post hoc analysis revealed a defect in the recorder but not interference of the ICD device. Two patients with LVADs had interference with capsule image acquisition while the capsule was in the small bowel. As best as can be determined from the localization hardware, these brief lapses (less than two minutes) seemed to occur when the capsule was in the upper abdomen closer to the LVAD.

## 4. Discussion

To our knowledge, this represents the largest study to date of CE studies performed in patients with IEDs. Others have demonstrated safety in *in vitro studies *[12, 13], and we have previously shown in very small studies that the performance of CE in patients with PMs and ICDs appeared to be safe [10, 11]. This study confirms the findings of those previous observations in a much larger group of patients.

 Other smaller retrospective studies are consistent with our observations. Bandorski et al. recently completed a retrospective study investigating the safety of CE in patients with PMs/ICDs, which similarly demonstrated the safety of CE in patients with cardiac devices [14]. Sixty-two patients (49-PM and 13-ICD) were identified via a standardized questionnaire sent out to high volume centers in Germany and Austria. Most patients (*N* = 58) had Given PillCam 2 capsule imaging but three patients had the Olympus EndoCapsule. Only 2 patients had gaps in the video or loss of images and none of the PMs or ICDs had impaired function. Unlike our patients, the majority of patients (*N* = 51) did not have their IEDs checked before and after the CE studies or (*N* = 49) undergo telemetry during their capsule studies, although all patients were observed clinically during the working battery time of their CE. This study did not include patients with LVADs. Two other smaller studies of twenty patients each with PMs or PMs/ICDs also have corroborated the safety of implantable devices and CE [13, 15]. Interestingly, Bandorski et al. and Cuschieri et al. both encountered instances where there was an interference between telemetry and CE with a loss of either some capsule images [14] or failure of the capsule localization device [15]. Neither of these difficulties was seen in our patients. 

 The importance of the safety data demonstrated in our study and those of others is increased when one considers that our population is aging. The elderly have an increased incidence of heart disease and the life threatening ventricular arrhythmias that accompany this morbidity, as well as an increased risk of gastrointestinal bleeding [16, 17]. There is also an increasing trend to see more ICDs used because cardiac trials have demonstrated a survival benefit in patients with life-threatening ventricular arrhythmias and other life-threatening arrhythmias [18]. ICDs also have a much more complex programmable function than a PM and thus a more extensive electronic circuitry, making it even more significant to be sure that CE is safe in this population. The patients requiring ICDs often have more advanced heart disease than patients with PMs alone. Maintaining the integrity of the ICD is fundamental because a disturbance of the device could alter several of its important functions. Alterations could cause (1) oversensing, which may deliver therapy when no arrhythmia existed; (2) undersensing, which could produce a failure to deliver therapy when it is needed; (3) pacemaker inhibition at a critical point such as aftershock delivery. 

 Recently, there has also been increasing use of LVADs, which are also complex and patients may have an association of increasing gastrointestinal bleeding from arteriovenous malformations or other GI lesions [19, 20]. LVADs have electronic circuits powered by an external battery and a mechanical component which propels blood. This adds yet another dimension to performing CE in this population [20] and also increases the need to be certain that CE can be safely used. It is presumed that LVAD interferes with image acquisition due to its size and location in the abdominal cavity. According to personal communication with a medical advisor at Given imaging, “gaps in capsule endoscopy are rare and may occur in patients without an IED” [21]. No Given data is available to really understand the increased rate of interference in patients with LVADs.

 As noted previously, there have been varying reasons for concern regarding the risk of interference of IEDs by external electromagnetic sources. This risk depends on (1) field strength of the electromagnetic source; (2) distance between the device and the electromagnetic source; (3) device parameters such as lead configuration (unipolar versus bipolar) and sensitivity [22]. The International Electro-technical Commission (IEC) Standard IEC 60601-1-2 (1993 Revision) is the international standard guiding radiofrequency immunity of medical devices. This standard sets a minimum immunity level of 3 volts per meter (V/m) in the 26–1000 MHz frequency range. ICDs require this degree of immunity at the CE frequency range of 439.09 MHz. There were no detectable alterations in programmed parameters suggesting that the “immunity threshold” of the ICD was not breached by the CE.

 No clinically significant alterations in the function of the PM and the multiple functions of the ICD/LVADs were revealed in pre- and postinterrogations. This includes undersensing and oversensing. This is likely due to rapid transit through the esophagus resulting in short duration exposure in the chest where the ICD/LVAD is generally situated. Additionally, the pacemaker antenna field radius is too small for the transmitter to cause interference. Therefore, no interference is seen with CE, and the vector of radiofrequency transmission is primarily within the abdomen and therefore in most instances away from the location of the ICD. This is an important factor in reducing the risk of interference for IEDs. 

However, with LVADs, particularly in the pediatric population being placed in the abdomen and even on rare occasions in children, PMs, this represents a potential risk for interference [23]. As this study demonstrates, the risk does not appear to be to the cardiac device but rather to the acquisition of capsule images. This suggests that the field strength of the electromagnetic source (i.e., LVAD) is sufficiently strong to interfere with image acquisition. This may alos occur because the size of the device is large and the field of influence is resultantly larger. The transit time of the capsule in the upper abdomen is also longer which may have been an additional factor causing transmitter interference. This would further suggest that the data recorder would have to be placed as far away as possible from the LVAD unit, a real constraint in patients with small body habitus. This study is not powered to detect the body mass index level at which point this interference represents a clear limitation. That would require a prospectively designed study. Even in patients with normal or higher BMI, when the capsule travels within the threshold field strength of the LVAD unit, image interference may potentially occur.

 From a practical point of determining a source of bleeding in these patients, we noted a lower percentage of patients (67%) with capsules reaching the cecum than that in our overall population of patients receiving CE where the completion rate was 75% [24]. We believe this occurred because many of the hospitalized inpatients were sicker and/or were on bed rest compared to patients receiving an ambulatory CE. Although not specifically analyzed, we speculate that hospitalized patients also may have had more acute bleeding or may have more comorbidities or even had on more medications such as narcotics. All of these factors may lead to either less mobile patients or less mobile gastrointestinal tracts. Since this observation was made [25], we have encouraged our hospital nursing staff placing the capsule to instruct patients who are able to be less sedentary and, thus, hopefully promote improved movement of the capsule through the gastrointestinal tract. Additionally, emerging technical developments with capsule endoscopes having longer recording times (12 hours versus 8 hours) should also help increase study completion rates. 

Overall, we believe that from our experience as well as that of other smaller studies that CE in patients with IEDs is a safe procedure. It does seem that concern should be focused toward LVADs and their potential to interfere with image acquisition of the capsule video. PMs and ICDs are often exclusively placed in the chest cavity while LVADs particularly in the pediatric population may be placed in the upper abdomen. We believe that this potential for interference can be overcome by making sure that the capsule leads are positioned as far away as possible from the LVAD to assure image acquisition. Additionally, this raises the issue of whether a certain band width reservation should be instituted for each group of devices to minimize or eliminate overlap interference. Future technological developments may make this concern for LVADs and even PM/ICDs a moot point but currently warnings are in effect and gastroenterologists should be cognizant of current guidelines for the use of CE in patients with implantable devices PM/ICDs.

## Figures and Tables

**Table 1:** Demographic data, indication, for capsule endoscopy, and study completion.

Patient demographics	*N* (%)
Number of capsule studies	118
Number of patients	108
Gender	
Male	68 (63)
Female	40 (37)
Average age	72.9 y/o (range 49–93)
Indications for capsule study	
Gastrointestinal bleeding	91 (77)
Anemia	17 (14)
Abdominal pain	6 (5)
Celiac disease	2 (2)
Diarrhea	1 (1)
Crohn's disease	1 (1)
Passage through ileocecal valve	79 (67)

**Table 2:** Device information and results of pre- and post-ICD interrogations.

Type of IED	*N* (%)
Pacemaker (PM)	74 (63)
ICD/ICD + PM	30 (25)
LVAD	8 (7)
LVAD + ICD	4 (3)
LVAD + PM	2 (2)
Pacemaker program	
DDD	7 (5.9)
DDDR	59 (50)
VVI	15 (12.7)
VVIR	22 (18.6)
DDI	4 (3.4)
DDIR	2 (1.7)
Missing data	9 (7.6)
Pre- and postinterrogation IED results	
Changes during study	None
Inappropriate ATP	None
Monitor	No arrhythmia
Inappropriate shocks	None
Inappropriate sensing	None

Abbreviations: IED: implantable electromedical device; PM: pacemaker; ICD: implantable cardiac defibrillator; LVAD: left ventricular assist device; DDD: dual chamber pacemaker with pacing and sensing in atrium and ventricle; DDDR: dual chamber pacemaker that is rate responsive; VVI: pacemaker that is ventricle paced, ventricle sensed, and pacemaker inhibited in response to a sensed beat; VVIR: (most commonly placed) ventricle paced, ventricle sensed, and pacemaker inhibited in response to a sensed beat and rate responsive; DD: atrial sensing and pacing, and ventricular sensing and pacing, but the pacemaker will not track intrinsic atrial activity; DDIR: atrial sensing and pacing, and ventricular sensing and pacing, but the pacemaker will not track intrinsic atrial activity and is rate responsive; ATP: antitachycardia pacing.
